# Exploring the Prospective Insights Into the Prognostics of N-terminal Pro-B-Type Natriuretic Peptide in Predicting Heart Failure Readmissions in a Tertiary Healthcare Setting

**DOI:** 10.7759/cureus.98142

**Published:** 2025-11-30

**Authors:** Ghulam Muhammad Shoaib, Uday Shree Akkala Shetty, Muhammad Zaman Baloch, Zahid Azam Chaudry

**Affiliations:** 1 Acute Medicine, Russells Hall Hospital, Dudley, GBR; 2 Internal Medicine, Southern Regional Medical Center, Riverdale, GEO; 3 Cardiology, Indus Medical College and Hospital, Tando Muhammad Khan, PAK; 4 Community Medicine, Nawaz Sharif Medical College, Gujrat, PAK

**Keywords:** heart failure, left ventricular ejection fraction, nt-probnp, prognosis biomarker, readmission

## Abstract

Background: Heart failure (HF) is a significant cause of morbidity and hospital readmission despite improvements in treatment. N-terminal pro-B-type natriuretic peptide (NT-proBNP) is a sensitive biomarker of ventricular wall stress and could be able to give prognostic insight into the post-discharge outcome. The aim of the study was to evaluate the prognostics of NT-proBNP in predicting heart failure readmissions in a tertiary healthcare setting.

Methods: This prospective observational study involved 200 patients (February 2025 to July 2025) with acute decompensated HF from the Department of Cardiology of Indus Medical College Hospital (IMCH) and Nawaz Sharif Medical College (NSMC), Pakistan. NT-proBNP was analyzed via electrochemiluminescence immunoassay (ECLIA) within 24 hours of discharge. The median NT-proBNP of the cohort was used to stratify the patients. T-tests and chi-square tests were used to compare demographic, clinical, and echocardiographic parameters. Multivariate Cox regression that was adjusted by renal function (eGFR), body mass index (BMI), atrial fibrillation, serum sodium, and congestion indicators was used to identify predictors of six-month readmission.

Result: NT-proBNP (mean) was measured as 2,410 ± 620 pg/mL and 8,920 ± 1,850 pg/mL in the lower and higher groups, respectively (p < 0.001). In total, readmission rates were 27% and higher in the high NT-proBNP group (40% vs. 14% p < 0.001). The higher group was found to have less left ventricular ejection fraction (LVEF) (38.6 ± 6.9% vs. 42.8 ± 7.2%, p = 0.001). Multivariate analysis established high NT-proBNP as a risk factor of readmission (heart rate (HR) = 2.76; 95% confidence interval (CI): 1.61-4.74; p < 0.001).

Conclusion: High NT-proBNP levels are a predictor of increased risk of HF readmission in six months. NT-proBNP measurement could help in risk stratification and focused follow-up; however, additional research is required to allow clinically validated cut-off values.

## Introduction

Heart failure (HF) is an increasing global health concern with a prevalence of more than 64 million people and a significant burden to cardiovascular morbidity and mortality [1]. Regardless of treatment progress, HF is a chronic, progressive syndrome that is accompanied by a significant number of exacerbations and frequent hospitalizations [2]. Readmission into the hospital after experiencing a period of decompensated HF is one of the main predictors of disease progression and healthcare cost, and about 20-30% of patients are readmitted within 30 days of discharge [3]. These readmissions not only deteriorate the survival outcomes but also cause enormous psychosocial and economic expenses to both patients and health systems [4].

Therefore, it is necessary to find good prognostic markers that could predict readmission earlier in order to maximize patient follow-up, treatment changes, and improve long-term outcomes [5]. N-terminal pro-B-type natriuretic peptide (NT-proBNP) is one of the biomarkers that have been found to have some clinical significance. It is a sensitive marker of hemodynamic compromise and is secreted by the ventricular myocytes in reaction to heightened stress on the walls, overload of the cardiac volume, and myocardial dysfunction [6].

NT-proBNP is widely used in diagnostic and prognostic stratification of acute and chronic HF [7]. Whereas some studies indicate that higher or continuously high NT-proBNP concentrations at discharge are linked with higher chances of readmission and poor outcomes [8], other studies have indicated inconsistent predictive soundness. One crucial factor that has contributed to this discrepancy is that most of the past analyses failed to properly consider the major determinants of NT-proBNP, including renal function, body mass index (BMI), and atrial fibrillation, which can significantly affect the levels of biomarkers and conceal their prognostic explanations [9]. In addition, the most appropriate cutoff value and timing to measure NT-proBNP to predict HF readmissions remain unclear, and thus further validation should be done in prospective studies [10].

This study aimed to determine the independent relationship between discharge NT-proBNP levels and six-month HF readmission in a prospective cohort by considering important clinical variables that are known to affect the NT-proBNP levels.

## Materials and methods

This prospective observational study involved 200 patients (February 2025 to July 2025) with acute decompensated HF from the Department of Cardiology of Indus Medical College Hospital (IMCH) and Nawaz Sharif Medical College (NSMC), Pakistan (Ref: MS37/03/2025) after obtaining informed consent within the framework of the Declaration of Helsinki. The OpenEpi version 3.01 (released 2013, Atlanta, GA, USA) was used to calculate the sample size based on a 95% confidence level, 80% power, and an expected readmission rate of 25% with at least 180 subjects [11]. A total of 200 patients participated in the study through a consecutive sampling method in order to consider the possibility of dropouts.

The inclusion criteria were patients aged 18 years or older who were admitted with acute decompensated heart failure (ADHF) and were discharged in a stable clinical state. Stability was characterized as no dyspnea at rest, no inotropic diuretics were required for at least 24 hours, and hemodynamic stability for ≥12 hours, systolic blood pressure ≥90 mmHg with no inotropic support, absence of active ischemia, and clinical euvolemia had been achieved through the assessment of jugular venous pressure, pulmonary auscultation, and stable peripheral edema. The study excluded patients having acute coronary syndrome, end-stage renal disease (estimated glomerular filtration rate (eGFR) < 30 mL/min/1.73 m^2^), severe hepatic dysfunction, active infection, malignancy, or incomplete NT-proBNP data.

At discharge, baseline demographic, clinical, biochemical, and echocardiographic parameters were documented. They were age, gender, BMI, weight, renal function (such as eGFR), serum sodium, comorbidities (diabetes, hypertension, and chronic obstructive pulmonary disease (COPD)), atrial fibrillation, congestion signs (jugular venous pressure (JVP)), left ventricular ejection fraction (LVEF), and discharge medications. Samples of venous blood were obtained within 24 hours prior to discharge. Samples were immediately processed to separate plasma, which was later stored at -80 °C till batch analysis. NT-proBNP was measured on the Cobas e601 analyses with a quantitative electrochemiluminescence immunoassay (ECLIA) (Roche Diagnostics, Germany). The analytical range of the assay was 5-35,000 pg/mL, and the intra- and inter-assay coefficient of variation was less than 5% and was calibrated using standards provided by the manufacturer.

The sample median NT-proBNP concentration was 5,100 pg/mL and was used to categorize patients into two groups. Group A consisted of patients with NT-proBNP levels of less than 5,100 pg/mL, and Group B consisted of patients with NT-proBNP levels more than 5,100 pg/mL. The median was chosen as the cut-off due to equal distribution among groups and the need to facilitate the exploratory analysis of discharge biomarker levels.

The duration of follow-up on patients was six months post-discharge. The hospital electronic records were used to identify readmissions and subsequently confirmed by structured telephone follow-up. The main study outcome was the time to first unplanned hospital readmission because of increasing HF.

The Shapiro-Wilk test is used to determine the normality of the continuous variables. Mean and standard deviation were used to show normally distributed data, the independent t-test was used to compare the data, frequencies and percentages were used to show categorical variables, and the Chi-square test was used to compare them. All baseline characteristics were analyzed using univariate Cox proportional hazards regression. The multivariate Cox model included variables with p < 0.10 in the univariate analysis or with clinical importance as predictors of NT-proBNP or HF outcomes, such as age, sex, diabetes, BMI, weight, eGFR, atrial fibrillation, serum sodium, LVEF, NT-proBNP, and congestion markers. Hazard ratios were adjusted, and the confidence intervals of these ratios were calculated. A statistical significance of p < 0.05 was considered. The analysis was done with the help of IBM SPSS Statistics for Windows, Version 26.0 (released 2018, IBM Corp., Armonk, NY).

## Results

Two hundred patients having ADHF were used, with 118 (59%) of these patients being male and 82 (41%) being female. The mean age was 62.7 ± 11.3 years. Patients were separated into two groups depending on the discharge NT-proBNP levels: Group A (NT-proBNP ≤ median, n = 100) and Group B (NT-proBNP > median, n = 100). NT-proBNP was used as a cutoff point to classify the groups at the median of 5,100 pg/mL.

Table 1 summarizes the baseline demographic, clinical, and laboratory characteristics of the research population. Group A and Group B did not have a significant difference in terms of age, sex distribution, hypertension, diabetes, ischemic etiology, BMI, weight, eGFR, serum sodium, atrial fibrillation, or congestion marker (jugular venous pressure ) (all p > 0.05). The LVEF of Group B was much lower than that of Group A (38.6 ± 6.9 vs. 42.8 ± 7.2, p = 0.001), which suggests more severe systolic dysfunction.

**Table 1:** Baseline demographics and clinical parameters (n = 200) *Statistically significant p < 0.05. BMI: body mass index, eGFR: estimated glomerular filtration rate, LVEF: left ventricular ejection fraction

Variable	Group A (NT-proBNP ≤ 5,100 pg/mL), n = 100	Group B (NT-proBNP > 5,100 pg/mL) , n = 100	Statistical test	Test value	p-value
Age (years, Mean ± SD)	61.9 ± 10.8	63.5 ± 11.9	t-test	1.00	0.32
Male gender, n (%)	60 (60.0)	58 (58.0)	χ²	0.08	0.78
Hypertension, n (%)	66 (66.0)	70 (70.0)	χ²	0.37	0.54
Diabetes mellitus, n (%)	52 (52.0)	56 (56.0)	χ²	0.29	0.59
Ischemic etiology, n (%)	63 (63.0)	68 (68.0)	χ²	0.52	0.47
BMI (kg/m²)	27.1 ± 4.2	27.8 ± 4.5	t-test	1.09	0.28
Weight (kg)	72.4 ± 12.3	74.1 ± 11.8	t-test	1.10	0.27
eGFR (mL/min/1.73 m²)	68.5 ± 15.2	66.2 ± 14.7	t-test	1.00	0.32
Serum sodium (mmol/L)	138.5 ± 3.6	138.1 ± 3.8	t-test	0.87	0.39
Atrial fibrillation, n (%)	20 (20.0)	26 (26.0)	χ²	0.94	0.34
Congestion marker, (JVP), n (%)	36 (36.0)	42 (42.0)	χ²	0.71	0.40
LVEF	42.8 ± 7.2	38.6 ± 6.9	t-test	3.40	0.001*

Table 2 reports the NT-proBNP levels and six months' readmission results. The mean NT-proBNP was 2,410 ± 620 pg/mL and 8,920 ± 1,850 pg/mL in Group A and Group B, respectively (p = 0.001). Total readmission was in 54 patients (27.0%). The readmission rate of Group B (40.0) was much higher than that of Group A (14.0; p < 0.001). Group B (median = 77 days) had a shorter time to readmission than Group A (median = 122 days).

**Table 2:** NT-proBNP levels and six-month readmission outcomes *Statistically significant p < 0.05. IQR: interquartile range

Variable	Group A (≤ median)	Group B (> median)	Test	Value	p-value
NT-proBNP (pg/mL, Mean ± SD)	2,410 ± 620	8,920 ± 1,850	t-test	27.1	<0.001*
Readmission within six months, n (%)	14 (14.0)	40 (40.0)	χ²	17.5	<0.001*
Time to readmission (days, median, IQR)	122 (98–145)	77 (55–102)	Log-rank	9.5	0.002*

The multivariate Cox proportional hazards regression was conducted to determine the predictors of six-month readmission (Table 3). The variables were age, sex, diabetes, BMI, weight, eGFR, atrial fibrillation, serum sodium, LVEF, congestion markers, and NT-proBNP higher than the median. NT-proBNP that exceeds the median was also significantly correlated with the risk of readmission (HR = 2.76; 95% CI: 1.61 4.74; p < 0.001). Reduced LVEF was also related to the risk of readmission (HR = 0.95/ 1% increment; 95% CI: 0.91-0.99; p = 0.01). The age, sex, diabetes, BMI, weight, eGFR, atrial fibrillation, serum sodium, and congestion markers were not significant in the multivariate model.

**Table 3:** Multivariate Cox regression analysis for the predictors of HF readmission *Statistically significant p < 0.05. HR: hazard ratio, CI: confidence interval, BMI: body mass index, eGFR: estimated glomerular filtration rate, LVEF: left ventricular ejection fraction, NT-proBNP: N-terminal pro-B-type natriuretic peptide

Variable	Hazard ratio (HR)	95% CI	p-value
Age (per year)	1.02	0.98–1.06	0.28
Male gender	0.91	0.53–1.56	0.73
Diabetes mellitus	1.34	0.77–2.33	0.29
BMI (kg/m²)	1.05	0.97–1.13	0.22
Weight (kg)	1.02	0.98–1.06	0.33
eGFR (mL/min/1.73 m²)	0.99	0.97–1.01	0.34
Atrial fibrillation, n (%)	1.21	0.72–2.04	0.46
Serum sodium (mmol/L)	0.97	0.90–1.04	0.37
Congestion markers, n (%)	1.18	0.74–1.89	0.48
LVEF (%)	0.95	0.91–0.99	0.01*
NT-proBNP > median	2.76	1.61–4.74	<0.001*

## Discussion

The present prospective cohort study investigated the prognostic capabilities of NT-proBNP level in the hospital readmission prediction of HF patients. The results of the current study indicated that the high levels of NT-proBNP at discharge were highly associated with a higher risk of readmission within six months. Patients with an NT-proBNP above the median had markedly reduced ejection fraction and six-month readmission rates than the patients with NT-proBNP below the median. The multivariate analysis has shown NT-proBNP to be an effective short-term prognostic biomarker in discharged patients, especially when corrected with significant confounders, including renal function (eGFR), BMI, atrial fibrillation, serum sodium, and congestion markers.

The correlation seen between the elevated NT-proBNP and risk of readmission is in line with the prior prospective studies, which have found NT-proBNP to be a high-quality surrogate endpoint of cardiac decompensation [12,13]. Previous studies have shown that patients with high post-discharge NT-proBNP levels are characterized by myocardial stress and subclinical volume overload, meaning that these patients are likely to experience relapse [14]. An investigation on comparative post-discharge monitoring approaches has shown that the use of NT-proBNP thresholds in the follow-up process enhances the early detection of at-risk patients [15]. These findings are corroborated by our observations, which indicate that the NT-proBNP measurement may be considered as a part of the discharge planning procedure in HF patients. The median-based threshold is, however, sample-specific and not externally validated, and therefore this approach has limited direct applicability to individual discharge planning.

It was previously stated that patients with high NT-proBNP were more susceptible to hypertension and diabetes mellitus, but the baseline features did not differ significantly between the high and low NT-proBNP groups and did not differ in the incidence of these comorbidities. LVEF among patients with increased NT-proBNP was significantly lower in relation to prior studies that have related NT-proBNP and systolic dysfunction [16]. High NT-proBNP can also indicate global cardiac stress as opposed to isolated systolic dysfunction, as evident in cohorts with a normal ejection fraction [17]. The reliability of NT-proBNP as a prognostic marker is enhanced with the inclusion of the main confounders in the multivariate analysis. These results demonstrate its possible use to predict patients at greater risk, perform personalized follow-up, and maximize therapy. However, the study is observational and, therefore, does not allow making judgments on the causation and efficiency of NT-proBNP-guided interventions [18].

This study has certain limitations. It was a one-center study with a very small sample, which can interfere with the generalizability of the findings. Although the prospective design and a well-defined endpoint are the strengths, several significant limitations are present. NT-proBNP levels also depend on age, renal function, BMI, atrial fibrillation, serum sodium, and congestion status, and even though these variables were now factored in the multivariate analysis, there is no external validation of the use of a median-based level, and, as a result, direct clinical applicability is limited. Patients were not stratified based on the HF phenotype (HFrEF vs. HFpEF), which can determine the NT-proBNP levels and risk of readmission. Moreover, earlier claims of higher incidences of hypertension and diabetes in the high NT-proBNP group were inaccurate, as no significant group differences were found. The cause of readmission was also not evaluated as HF exacerbation. Further multicentric studies based on large populations, extended follow-up, and specific phenotype analyses should be applied to enhance the validation of the prognostic value of NT-proBNP.

## Conclusions

This study demonstrates that higher levels of NT-proBNP at discharge help to identify patients with ADHF who are more prone to readmission within six months. Higher NT-proBNP levels also demonstrated reduced LVEF among patients with higher NT-proBNP levels, and this is indicative of the prognostic nature of this biomarker in predicting cardiac stress and dysfunction.

Although NT-proBNP is useful in risk stratification, the observational design and sample-specific median threshold are restrictive to the direct clinical applicability, and causality is not inferred. Larger population, protracted follow-ups, and phenotype-based studies should be conducted in future multicenter studies to confirm NT-proBNP cutoff points and inform individualized post-discharge care.
